# Correlation of APRIL with production of inflammatory cytokines during acute malaria in the Brazilian Amazon

**DOI:** 10.1002/iid3.208

**Published:** 2018-01-03

**Authors:** Raquel A. Pinna, Adriana C. dos Santos, Daiana S. Perce‐da‐Silva, Luciene A. da Silva, Rodrigo N. Rodrigues da Silva, Marcelo R. Alves, Fátima Santos, Joseli de Oliveira Ferreira, Josué C. Lima‐Junior, Déa M. Villa‐Verde, Paula M. De Luca, Carla E. Carvalho‐Pinto, Dalma M. Banic

**Affiliations:** ^1^ Laboratory of Clinical Immunology, Oswaldo Cruz Institute Oswaldo Cruz Foundation Avenida Brasil 4365, Manguinhos Rio de Janeiro, RJ Brazil 21040‐360; ^2^ Laboratory of Experimental Pathology, Institute of Biology Fluminense Federal University Niterói, RJ Brazil 24020‐140; ^3^ Laboratory of Imunoparasitology Research, Oswaldo Cruz Institute Oswaldo Cruz Foundation Avenida Brasil 4365, Manguinhos Rio de Janeiro, RJ Brazil 21040‐360; ^4^ Laboratory of Research in Pharmacogenetics, National Institute of Infectology Oswaldo Cruz Foundation Avenida Brasil 4365, Manguinhos Rio de Janeiro, RJ Brazil 21040‐360; ^5^ Laboratory of Entomology, LACEN/RO Rua Anita Garibalde 4130 − Costa e Silva Porto Velho, RO Brazil 76803‐620; ^6^ Laboratory on Thymus Research, Oswaldo Cruz Institute Oswaldo Cruz Foundation Avenida Brasil 4365, Manguinhos Rio de Janeiro, RJ Brazil 21040‐360

**Keywords:** APRIL/BAFF, malaria, TACI

## Abstract

**Introduction:**

A proliferation‐inducing ligand (APRIL) and B cell activation factor (BAFF) are known to play a significant role in the pathogenesis of several diseases, including BAFF in malaria. The aim of this study was to investigate whether APRIL and BAFF plasma concentrations could be part of inflammatory responses associated with *P. vivax* and *P. falciparum* malaria in patients from the Brazilian Amazon.

**Methods:**

Blood samples were obtained from *P. vivax* and *P. falciparum* malaria patients (*n* = 52) resident in Porto Velho before and 15 days after the beginning of treatment and from uninfected individuals (*n* = 12). We investigated APRIL and BAFF circulating levels and their association with parasitaemia, WBC counts, and cytokine/chemokine plasma levels. The expression levels of transmembrane activator and calcium‐modulating cyclophilin ligand interactor (TACI) on PBMC from a subset of 5 *P. vivax*‐infected patients were analyzed by flow cytometry.

**Results:**

APRIL plasma levels were transiently increased during acute *P. vivax* and *P. falciparum* infections whereas BAFF levels were only increased during acute *P. falciparum* malaria. Although *P. vivax* and *P. falciparum* malaria patients have similar cytokine profiles during infection, in *P. vivax* acute phase malaria, APRIL but not BAFF levels correlated positively with IL‐1, IL‐2, IL‐4, IL‐6, and IL‐13 levels. We did not find any association between *P. vivax* parasitaemia and APRIL levels, while an inverse correlation was found between *P. falciparum* parasitaemia and APRIL levels. The percentage of TACI positive CD4+ and CD8+ T cells were increased in the acute phase *P. vivax* malaria.

**Conclusion:**

These findings suggest that the APRIL and BAFF inductions reflect different host strategies for controlling infection with each malaria species.

## Introduction

Malaria is one of the most important human parasitic diseases. Nearly half the world's population is at risk of contracting malaria, with an estimate global annual incidence of about 212 million clinical cases and almost 429,000 deaths 1. Among the five *Plasmodium* species that infect humans, *P. falciparum* and *P. vivax* are responsible for 95% of malaria cases around the world. Although *P. falciparum* accounts for the vast majority of morbidity and mortality, *P. vivax* has a wider geographic distribution and causes significant symptomatic disease 2. Currently, there is no available vaccine to prevent malaria. Although sterile immunity against malaria parasite is most likely never achieved, individuals living in malaria‐endemic areas can acquire a state of clinical immunity towards severe illness and death. The mechanisms underlying the development of semi‐immunity are not entirely understood. However, it is well established, that naturally acquired immunity against blood stage parasite involves both CD4+ T cells and antibodies 3. The importance of antibodies was recognized in the studies demonstrating that passive transfer of serum Immunoglobulin G (IgG) from clinically immune individuals into non‐immune recipients substantially reduced parasite burden and the following clinical symptoms 4, 5, 6. Furthermore, many studies showed that the quality, level, and breadth of the antibody response are critical components of malaria clinical immunity 7, 8, 9, 10, 11, 12. However, malaria clinical immunity develops slowly and is ineffectively maintained, suggesting a poor generation of protective immune memory. This happens are attributable to a number of different factors, which include the disturbance of immune homeostasis by *Plasmodium* spp. 13, 14. At B‐cells level, alterations such as polyclonal B‐cell activation, atypical memory B‐cell expansion, and deletion of specific B‐cell subsets are well described in the context of malaria 13, 15, 16, 17, 18, 19, 20. However, the mechanisms leading to this B‐cell dysregulation are not entirely understood. Studies indicate that the members of the tumor necrosis factors (TNF) superfamily such as B cell activation factor (BAFF; also known as BlyS) and a proliferation‐inducing ligand (APRIL) have an important role in the T‐cell independent antibody production, immunoglobulin isotype switching and in the selection, maturation and survival of B cells 21, 22. In addition, BAFF drives the expansion of Th1 and Th17 pathways which increase Th1‐associated inflammatory responses 23. Both cytokines are produced by a variety of cell types, particularly leukocytes, and share two surface receptors expressed on B cells; transmembrane activator and calcium modulator and cyclophilin ligand interactor (TACI) and B‐cell maturation antigen (BCMA) 24. TACI expression is restricted to B cells and a subset of activated T cells 21.

The mechanisms regulating BAFF and APRIL‐system molecule expression are poorly known. However, it is recognized that cytokines such as interferon (IFN)‐γ, IFN‐α, TNF, and Interleukin (IL)‐10 as well as granulocyte colony‐stimulating factor (G‐CSF), CD40 ligand (CD40‐L), lipopolysaccharides, and peptidoglycans can upregulate BAFF or APRIL expression in different cells 23, 25, 26, 27, 28, 29, 30.

In the recent years, several studies have been shown that BAFF, APRIL, and their receptors play a significant role in the pathogenesis of various noninfectious and infectious diseases 23, 26, 31, 32, 33, 34, 35, including BAFF in malaria 18, 35, 36. In *P. falciparum* malaria, BAFF levels are increased in plasma samples from infected children in Kenya 36 and in placental tissue from infected pregnant women in Tanzania 37, which correlate with disease pathogenesis and severity. Moreover, in vitro, both the soluble fraction of *P. falciparum* antigens and hemozoin enhanced BAFF surface expression as well as secretion by human monocytes and increased B cell proliferation and IgG secretion 38. While there is some evidence indicating a pathogenic role of BAFF in *P. falciparum* malaria, as before mentioned, the role of APRIL in malaria is still unknown.

Brazil has a peculiar malaria epidemiological situation, in which *P. vivax* accounts for more than 85% of all malaria cases 39. Furthermore, the dynamics of malaria infection and severity differ greatly from malaria endemic regions of Africa and Asia 1. Therefore, the present study investigated whether BAFF and APRIL plasma concentrations could be part of inflammatory response associated with uncomplicated *P. falciparum* and *P. vivax* malaria in some adults from the Brazilian Amazon who were followed up during the acute and convalescent phases of infection.

## Materials and Methods

### Study area and subjects

**Figure 1:**
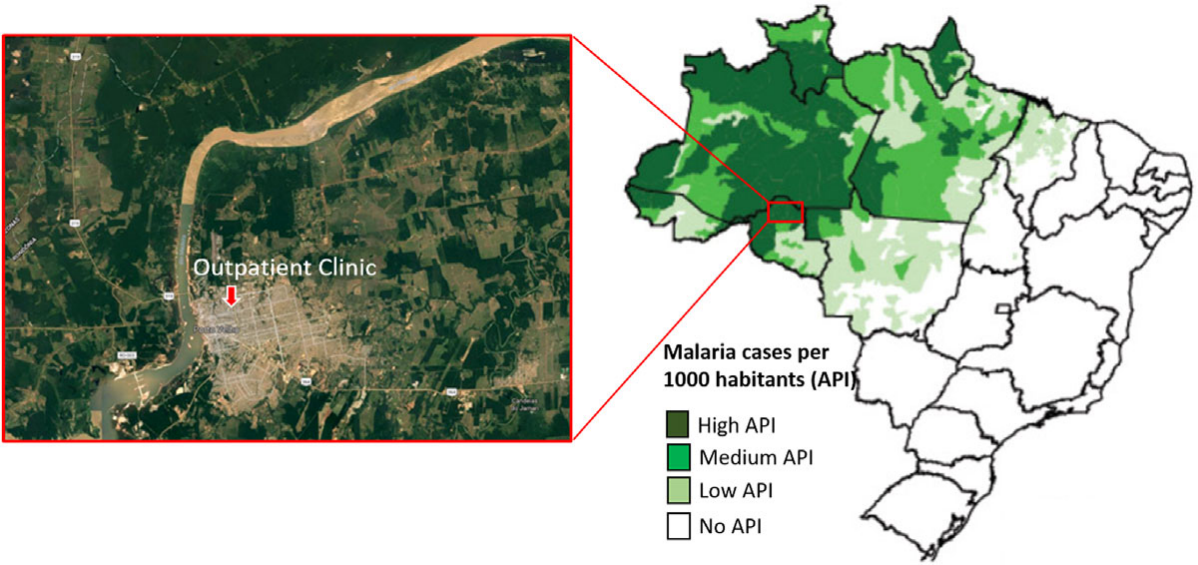
Map showing the malaria endemic area—Porto Velho municipality, Rondônia State, Brazil.

The study was conducted in 52 patients with non‐complicated *P. vivax* (*n* = 33) or *P. falciparum* (*n* = 19) malaria who resided in the communities of Porto Velho, the capital of the Rondônia state (8°45′43″ S, 63°54′14″ W), in the Brazilian Amazon malaria endemic region (Fig. 1). Transmission of malaria in Rondônia is present throughout the year with seasonal fluctuations and maximum transmission occurring during the dry season between April and September 40, a period in which our sample and survey data were collected.

This population is composed of natives and Brazilian migrants inhabiting this area for variable times since the 1970s (average length of residence in malaria endemic area media = 27.5 ± 12 years, median = 26.5, IQR = 20.0–34.5). All malaria patients who were enrolled in this study complied with the following criteria: 1) they presented symptoms; 2) they had thin‐thick blood smears positive for malaria parasites; 3) they were not indigenous; 4) they were not prisoners; 5) they were all over 12 years old; and 6) no females were pregnant or breast‐feeding.

The patients sought health care at the government outpatient clinic in Porto Velho after the onset of malaria symptoms. Blood samples were collected on the day of diagnosis before treatment (in the acute phase = D0) and 15 days later (in the convalescence phase = D15). After giving written informed consent which was reviewed and approved by the Ethical Committee of the Oswaldo Cruz Foundation (354/06), questionnaires were administered inquiring about demographic data, time of residence in the endemic area, personal and family malaria histories, presence of malaria symptoms, and use of malaria prophylaxis for each survey participant. After blood collection, donors positive for *P. vivax* and/or *P. falciparum* were subsequently treated according to Brazilian Ministry of Health standards for malaria therapy: for uncomplicated malaria is recommended chloroquine combined to primaquine to *P. vivax* infection and artemether‐lumefantrine (Coartem®) to *P. falciparum* infection. Asexual blood forms of *P. vivax* and *P. falciparum* were cleared from the peripheral blood of all patients included in the study following therapy and no parasite reappearance was observed during follow‐up. The control group (*n* = 12) was composed of apparently healthy individuals who lived in the same geographic area, but were negative for malaria parasites as assessed by thick blood films and had not reported any malaria episodes for at least 5 years.

### Sample collection, malaria diagnosis, and peripheral blood mononuclear cells isolation

Venipuncture drew venous blood samples (5 mL) in EDTA‐containing tubes. Thin and thick blood smears of all donors were examined for malaria parasites. Parasitological evaluations were done by examination of 200 fields at 1,000‐fold magnification under oil‐immersion. The number of parasites was counted against 200 white blood cells (WBC). The parasite density per microliter of blood was calculated by multiplying the number of parasites counted by number of WBC divided by 200 41. If less than 9 parasites were detected, 300 additional leukocytes were counted to obtain more precise results. To increase the sensitivity of parasites detection, molecular analyses using specific primers for genus (*Plasmodium* sp.) and species (*P. falciparum* and *P. vivax*) were performed in all samples as previously described 42. Subjects were considered to have malaria if they were positive in thick blood smear and/or in Polymerase Chain Reaction (PCR).

WBC count was performed in the acute and during the convalescence phase using an automatic hematology analyzer (Pentra ABX). The cell counters provided data on leukocyte, lymphocyte, eosinophil, neutrophil, band cell, monocyte, and basophil counts. Qualified pathologists examined the smears. Of the subjects who agreed, venous peripheral blood (10 mL) was collected in heparin‐containnig tubes for flow cytometry analysis (five patients and four controls). Peripheral blood mononuclear cells (PBMC) were separated from whole blood by density gradient centrifugation (Ficoll‐Hypaque; density 1.077 g/mL; Sigma Company, USA). Cell viability was determined using the trypan blue dye exclusion assay. Cells were resuspended to a concentration of 2 × 10^7^cells/mL in the freezing medium consisting of 90% heat‐inactivated fetal bovine serum (FBS; Hyclone, USA) and 10% dimethyl sulfoxide (DMSO; Sigma, USA) and placed into Nalgene cryogenic freezing container (Nalgene Labware). The freezing container was then stored at −70°C overnight, and frozen cell samples were transferred to liquid nitrogen container (−196°C).

### Multiplex microsphere cytokine and chemokine immunoassay

Cytokine and chemokine concentrations in plasma samples were determined by Luminex technology (Luminex Corporation, Austin, TX, USA). Fourteen cytokines IL‐1β, IL‐2, IL‐4, IL‐5, IL‐6, IL‐7, IL‐10, IL‐12 p70, IL‐13, IL‐17, IFN‐γ, TNF‐α, G‐CSF, granulocyte‐macrophage colony‐stimulating factor (GM‐CSF), and three chemokines (IL‐8, MCP‐1, and MIP‐1β) were analyzed using a BioPlex‐Kit assay (Bio‐Rad Laboratories, Hercules, CA, USA). The assay was performed according to the manufacturer's instructions using a BioPlex‐kit in combination with the Luminex system. Briefly, 50 μL of standard or test sample along with 50 μL of mixed beads were added into the wells of a pre‐wetted 96‐well microtitre plate. After 1 h of incubation and washing, 25 μL of detection antibody mixture was added and the samples were incubated for 30 min and then washed. Finally, 50 μL of streptavidin‐PE was added and after 10 min of incubation and washing, the beads were resuspended in 125 μL assay buffer and analyzed using a BioPlex suspension array system (Bio‐Rad Laboratories) and the Bio‐Plex manager software (v.3.0). A minimum of 100 beads per region were analyzed. A curve fit was applied to each standard curve according to the manufacturer's manual and sample concentrations were interpolated from the standard curves. The limit of cytokine detection using this method was 2 pg/mL for all cytokines and chemokines. The median cytokine and chemokine levels in 12 healthy controls were 2 pg/mL for IL‐2, IL‐4, IL‐5, IL‐6, IL‐7, IL‐10, IL‐12 p70, IL‐13, IL‐17, and GM‐CSF, 5.85 pg/mL for IL‐1β, 17.64 pg/mL for IFN‐γ, 23.23 pg/mL for TNF‐α, 4.40 pg/mL for G‐CSF, 379.13 pg/mL for MCP‐1, and 994.8 pg/mL for MIP‐1β.

### APRIL and BAFF ELISA

The plasma concentrations of APRIL and BAFF were measured with Human APRIL platinum ELISA Kit (Bender MedSystems GmbH, Vienna, Austria) and Human BAFF Instant ELISA kit (Bender MedSystems GmbH, Vienna, Austria) according to the manufacturer's instructions. Assays were performed in duplicate on 1/2 diluted samples. Plasma APRIL and BAFF levels were quantified using the in‐Kit standard curves for the respective cytokines. The minimal detectable doses of APRIL and BAFF in ELISA assay were 1.43 and 3.03 ng/mL, respectively. The median APRIL levels in 12 healthy controls were 5.02 ng/mL and the median BAFF were 6.72 ng/mL.

### Flow cytometry

Cryopreserved PBMC were thawed at 37°C, and Benzonase‐supplemented complete medium was slowly added (90% RPMI Medium 1640—GIBCO/USA, 10% Fetal bovine serum—HyClone/USA and Benzonase Nuclease—Sigma–Aldrich, USA, to a final concentration of 50 U/mL) 43. The cells were washed and resuspended in medium without Benzonase twice. Viability and recovery were measured using trypan blue exclusion dye. For immunophenotypic analysis, 500,000 to 1,000,000 viable cells were transferred into a 96‐well U‐bottom plate, washed once with 200 μL PBS, and incubated with 100 μL of PBS/milk buffer containing 10% of human AB serum for 15 min. Cells were then washed with PBS/BSA buffer (PBS containing 0,1% BSA), and stained with 50μL of an antibody mixture comprising CD19‐APCCy5 (e‐Bioscience, clone SJ25‐CI), CD5‐FITC (eBioscience, clone L17F12), TACI/TNFRSF13B‐PE (R&D Systems, clone 165604), CD3‐APCCy7 (BioLegend, clone HIT3a), CD4‐PECy7 (e‐Bioscience, clone OKT4), and CD8‐PE Texas Red (Invitrogen, clone 3B5)) in PBS/BSA buffer for 45 min at 4°C. Cells were washed twice in PBS/BSA buffer and fixed with 10% paraformaldehyde. After another wash step, the cells were resuspended in 500 μL of PBS/BSA buffer. All samples were live gated by side and forward scatter on lymphocytes, and 10,000 to 20,000 events were acquired inside the lymphocytes gate in a Cyan ADP flow cytometer (Dako, USA). Flow cytometry data were analyzed later using FlowJo v.10.0.8 software (USA).

### Statistical analysis

The survey data were recorded and entered into a database created with EPI Info 2007 (Centers for Disease Control and Prevention, Atlanta, GA, USA). Statistical analysis was carried out with GraphPad Prism 5.0 (GraphPad Software Inc., Chicago, IL, USA) and R software. We previously checked whether the data was normally distributed by performing the D'Agostino's and Pearson omunibus normality test. As the data was not normally distributed we used Wilcoxon matched‐paired test to calculate significance levels between patients (D0 and D15) and Mann–Whitney unpaired *T* test to calculate significance levels between patients and control group. Spearman‐rank correlation analysis was used to calculate correlations. Reported *P* values are two‐tailed, and statistical differences were considered significant when *P* values were less or equal to 0.05.

## Results

### General features of the study population

General features of the volunteers are displayed in Table 1. The median of age, period living in endemic area and number of previous malaria were similar between the *P. vivax* and *P. falciparum* malaria patient groups. Although the median of parasitemia appeared to be higher in *P. vivax* than in *P. falciparum* infected patients, this difference was not statistically significant. All patients were symptomatic at the time of enrollment independent of *Plasmodium* species and the most common symptoms reported by them were fever (91%), headache (89%), myalgia (77%), chills (77%), and vomiting (64%). However, no correlation was found between parasitaemia and axillary temperature (*P* < 0.05). There was no significant difference among the general features of the patient groups and control group, except for the absence of circulating parasites and the lowest number of previous malaria in the control group. All patients were parasitemia negative by day 15 of follow‐up after receiving effective drug treatment.

**Table 1:** General features of the study population

Parameter	Control (*n* = 12)	*P. vivax* (*n* = 33)	*P. falciparum* (*n* = 19)
Gender (%)			
Male	42	73	74
Female	58	27	26
Age (years)	29 (24.5–40)	28 (22–36.5)	28 (23–42)
Parasitaemia (number ofparasites/μL)	–	3,200 (1,047–7,758)	1,400 (890–3,338)
Years of residence in malaria endemic area	29 (18.25–32)	25 (22–34)	25 (19–38)
Previous malaria episodes	0 (0–2.25)	4 (1–10)**	3 (1–10)*

Data in the table present median (25th and 75th percentile) for each parasitological or epidemiological parameter. The Mann–Whitney test was used to compare groups. (*) *P* = 0.01 and (**) *P* = 0.009 between indicated infected group and control.

### Increased levels of APRIL and BAFF in the acute phase of malaria

We analyzed APRIL and BAFF plasma levels from 12 healthy controls and 52 patients infected with *P. vivax* (*n* = 33) or *P. falciparum* (*n* = 19). To investigate changes in APRIL and BAFF levels during the infection, we compared levels in the plasma from the same patient during the acute (D0) and convalescent (D15) phases.

**Figure 2:**
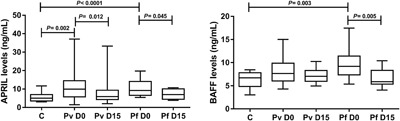
Plasma levels of a proliferating‐inducing ligand (APRIL) and B‐cell activating factor (BAFF) in the control and malaria groups. **A:** APRIL levels in control (*n* = 12), *P. vivax* (*n* = 33), and *P. falciparum (n* = 19) patients on the day of diagnosis and 15 days later; **B:** BAFF levels in control, *P. vivax* and *P. falciparum* patients on the day of diagnosis and 15 days later. Each box represents 25th and 75th percentiles. Lines inside the boxes represent median values. Lines outside the boxes represent the minimum and maximum values. C, control; Pv, *P. vivax*; Pf, *P. falciparum*; D0, the day of diagnosis; D15, 15 days after diagnosis. Mann– Whitney test and Wilcoxon matched‐paired test were used to calculate significance levels between two groups.

As shown in Figure 2A, APRIL plasma levels were significantly higher in *P. vivax* (median = 9.93 ng/mL, IQR = 5.73–14.47, *P* = 0.002) and *P. falciparum* (median = 9.15 ng/mL, IQR = 6.52–14.04, *P *< 0.0001 patients during acute phase (D0) than those of the controls (median = 5.02 ng/mL, IQR = 3.48–6.93). To investigate changes in APRIL levels during the infection, we compared APRIL levels in the plasma from the same patient during the acute (D0) and convalescent (D15) phases. During the convalescent phase *P. vivax* (median = 5.85 ng/mL, IQR = 4.26–9.29, *P* = 0.012) and *P. falciparum* (median = 6.93 ng/mL, IQR = 4.39–10.03, *P* = 0.045) patients had significantly lower levels of APRIL than during the acute phase. The levels of APRIL were similar between *P. vivax* and *P. falciparum* patients during both acute and convalescent phases (*P* *>* 0.05, for all).

As shown in Figure 2B, BAFF plasma levels were detected at high concentrations in *P. falciparum* patients (median = 9.20 ng/mL, IQR = 7.36–11.43, *P* = 0.003) during the acute phase and returned to the levels similar to those of the controls (median = 6.72 ng/mL, IQR = 4.86–7.80). During the convalescent phase, *P. falciparum* (median = 5.89, IQR = 5.36–8.32, *P* = 0.005) patients had significantly lower levels of BAFF than during the acute phase. In *P. vivax* patients, although the median BAFF levels were increased during the acute (median = 7.63 ng/mL, IQR = 6.04–9.87) and convalescent (median = 7.06 ng/mL, IQR = 5.95–8.30) phases, these levels were not statistically different from those of the controls (*P* *>* 0.05, for both). The levels of BAFF were similar between *P. vivax* and *P. falciparum* patients during both acute and convalescent phases (*P > *0.05, for all). Furthermore, no correlation was found between APRIL and BAFF plasma levels during both acute and convalescent phases of *P. vivax* (*r* = 0.245, *P* = 0.296; *r* = −0.103, *P* = 0.665, D0 and D15, respectively) and *P. falciparum* (*r* = −0.099, *P* = 0.739; *r* = 0.013, *P* = 0.964, D0 and D15, respectively) malaria. The correlation between APRIL plasma levels and parasitaemia was only observed in *P. falciparum* malaria (*r* = −0.500, *P = *0.031). There was no correlation between BAFF plasma levels and parasitaemia in *P. falciparum* (*r* = −0.169, *P* = 0.562) or *P. vivax* malaria (*r* = 0.104, *P* = 0.663).

### No correlation between APRIL/BAFF plasma levels and WBC counts during the acute or convalescent malaria phases

White blood cell counts showed significant changes in *P. vivax* and *P. falciparum*‐infected patients (Table 2). As expected, a significant decrease in total white blood cells, lymphocytes, and eosinophils were evident in *P. vivax* and *P. falciparum* malaria acute phase in comparison to control group but returned to normal levels in the convalescent phase. Band cell counts, on the other hand, were enhanced in *P. vivax* and *P. falciparum* malaria patients during the acute phase and also returned to normal levels in the convalescence. We assessed the relationship between APRIL/BAFF levels and WBC counts in healthy controls and malaria patients. However, no correlation was found between any of the WBC counts evaluated and the APRIL or BAFF plasma levels during the acute or convalescent malaria phases, as well as in the healthy control group.

**Table 2:** Leukogram profile of control and malaria patient groups in the acute (D0) and convalescent (D15) phases of infection

	Control (*n* = 12)	*P. vivax* (*n* = 33)		*P. falciparum* (*n* = 19)	
Leukogram (cell/μL)	D0	D0	D15	D0	D15
Leukocytes	6,400 (5,650–7,550)	5,100 (3,850–6,600)^b^	5,400 (4,950–6,625)	4,700 (3,900–6,400)^ae^	6,200 (4,675–7,500)
Neutrophils	3,842 (3,090–4,572)	3,468 (2,155–4,459)	2,955 (2,349–3,432)	3,420 (1,980–4,221)	3,018 (2,174–3,744)
Band cells	0 (0–0)	62 (0–399)^bef^	0 (0–0)	152 (47–474)^ce^	0 (0–0)
Lymphocytes	2,160 (1,770–2,515)	1,160 (795–1,463)^ce^	2,014 (1,582–2,246)	1,152 (714–1548)^ce^	1,989 (1,581–2,936)
Monocytes	328 (233–525)	371 (182–512)	322 (222–468.8)	363 (325–553)	308 (225.8–394.5)
Eosinophils	152 (67.5–249)	62 (36.5–136)^ae^	298.5 (122.5–393)	80 (33–405)^e^	246.5 (132.5–766.5)
Basophils	0 (0–0)	0 (0–0)	0 (0–0)	0 (0–0)	0 (0–0)

Data in the table present median (25th and 75th percentile) for each WBC evaluated in control and malaria‐infected groups. a) *P *< 0.05, b) *P *< 0.01 and c) *P *< 0.001 between indicated infected group and control, respectively—Mann–Whitney test; d) *P *< 0.05 and e) *P *< 0.001 between the acute and the convalescent phases—Wilcoxon matched‐paired test; f) *P *< 0.05 between *P. vivax* and *P. falciparum* infected groups—Mann–Whitney test. D0: day of diagnosis. D15: 15 days after diagnosis.

### Correlation between APRIL and inflammatory cytokines plasma levels during the acute phase of *P. vivax* malaria

Table 3 shows the plasma cytokine and chemokine levels in acute and convalescent phase studied subjects. Patients infected with *P. vivax* or *P. falciparum* presented an increase in IFN‐γ, TNF‐α, IL‐6, IL‐8, IL‐10, IL‐13, MIP1β, and G‐CSF levels during the malaria acute phase. *P. falciparum* infected individuals also presented enhanced concentration of IL‐2 in plasma during the acute stage. To evaluate changes in cytokine concentrations during the infections, we compared patients from the same infected group during the acute (D0) and convalescent (D15) phases. *P. vivax* and *P. falciparum* patients presented higher levels of IFN‐γ, TNF‐α, IL‐6, IL‐8, IL‐17, and MIP1β in the convalescent phase than in the acute phase. Although *P. vivax* and *P. falciparum* malaria patients have similar cytokine profiles during infection, *P. falciparum* patients presented higher levels of IFN‐γ and TNF‐α, in acute phase and of IFN‐γ, IL‐6, IL‐8, IL‐17, and G‐CSF in convalescent phase than *P. vivax* patients. Moreover, the levels of IL‐2, IL‐4, and IL‐12 were increased in the convalescent phase of *P. falciparum* infection. On the contrary, IL‐10 levels decreased in the *P. vivax* and *P. falciparum* malaria convalescent phase and returned to healthy normal levels. The cytokines IL‐5, IL‐7, and GM‐CSF were not detectable in most plasma samples. No differences were observed in MCP‐ 1 levels compared with controls. The lL‐10 and G‐CSF levels were positively correlated with parasitaemia in the *P. vivax* (*r* = 0.563, *P* = 0.0006; *r* = 0.430, *P* = 0.01, respectively) and *P. falciparum* (*r* = 0.428, *P* = 0.037; *r* = 0.657, *P* = 0.002) malaria acute phase. The same was true for IFN‐γ (*r* = 0.518, *P* = 0.02), IL‐2 (*r* = 0.611, *P* = 0.005) and IL‐4 (*r* = 0.655, *P* = 0.002) in *P. falciparum* malaria patients.

**Table 3:** Cytokine and chemokine profiles of control and malaria patient groups in the acute (D0) and convalescent (D15) phases of infection

		*P. vivax* (*n* = 33)		*P. falciparum* (*n* = 19)	
Plasma levels pg/mL	Control (*n* = 12)	D0	D15	D0	D15
IFN‐γ	17.64 (17.26–18.87)	58.96 (39.12–243.3)^c^	113.4 (63.27–393.2)^cd^	142.8 (2–336.2)^cg^	440.7 (137.9–527.3)^cdg^
TNF‐α	23.23 (17.11–36.41)	60.14 (39.68–148.8)^a^	328.4 (178.6–607)^cf^	192.7 (51.8–335.1)^bg^	404.6 (204.6–749.1)^cd^
IL‐1	5.85 (5.59–6.57)	10.45 (3.84–15.49)	14.76 (12.16–24.54)	13.63 (11.09–18.63)	21.00 (16.31–25.31)
IL‐2	2 (2–2)	2 (2–250.4)	9.05 (2–370.3)	154.6 (2–337.5)^a^	438.7 (83.81–538.3)^bd^
IL‐4	2 (2–2)	2 (2–2)	2 (2–225.3)	2 (2–123)	222.2 (2–364.5)^ad^
IL‐6	2 (2–2)	152.1 (2–827.7)^b^	460.7 (117.7–2,197)^cd^	335.9 (238.3–1,552)^b^	2,974 (1,331–3,563)^ceg^
IL‐8	47.01 (6.33–259.5)	959.2 (249.3–3,367)^a^	5,440 (3,528–14,881)^cf^	2,457 (819.8–8,042)^bg^	16,506 (12,263–22,161)^cfh^
IL‐10	2 (2–2)	1753.51 (461.8–3,763)^cf^	2 (2–2)^g^	1,627 (795.3–3,613)^cf^	2 (2–183.7)
IL‐12	2 (2–2)	2 (2–2)	2 (2–152.7)	2 (2–112.7)	219.8 (2–234.1)^bdg^
IL‐13	2 (2–2)	66.79 (2–301)^a^	34.21 (2–286)	301 (270.3–342.8)^bg^	301 (203.2–329.3)^ag^
IL‐17	2 (2–2)	2 (2–89.3)	123.2 (2–239.7)^be^	2 (2–154.3)	279.3 (163.8–362.1)^cfg^
G‐CSF	4.4 (4.23–4.56)	11.07 (7.16–48.41)^b^	16.89 (12.16–57.41)^b^	11.11 (2–67.99)^e^	96.45 (33.94–113.1)^cg^
MCP1	379.1 (294.8–574.4)	815.7 (97.24–3,241)	234.8 (76.14–848.8)	788.5 (559–1,945)	720.6 (360.2–957.4)
MIP1β	994.8 (416.4–2008)	5,641 (3,272–9,833)^b^	11,685 (8,370–26,566)^cf^	7,506 (5,027–11,697)^c^	14,601 (12,435–21,855)^cf^

Data in the table present median (25th and 75th percentile) for cytokines and chemokines in control and malaria‐infected groups. a) *P *< 0.05, b) *P *< 0.01 and c) *P *< 0.001 between indicated infected group and control, respectively—Mann–Whitney test; d) *P *< 0.05, e) *P *< 0.01 and f) *P *< 0.001 between the acute and the convalescent phase—Wilcoxon matched‐paired test; g) *P*< 0.05 and h) *P *< 0.01 between *P. vivax* and *P. falciparum* infected groups—Mann–Whitney test. D0: day of diagnosis. D15: 15 days after diagnosis.

As shown in Table 4, in *P. vivax* acute phase, APRIL plasma levels were positively correlated with IL‐ 1 (*r* = 0.409, *P* = 0.018), IL‐2 (*r* = 0.386, *P* = 0.026), IL‐4 (*r* = 0.396, *P* = 0.022), IL‐6 (*r* = 0.365, *P* = 0.037), and IL‐13 (*r* = 0.352, *P* = 0.045) while BAFF plasma levels were not statistically correlated with any of the cytokines/chemokines assayed. No correlation was observed between APRIL, BAFF, and cytokine plasma levels during the *P. vivax* convalescent phase. No relationship was found between any of the cytokines/chemokines assayed and the APRIL or BAFF plasma levels in *P. falciparum* acute or convalescent phases and control group (*P* *>* 0.05, for all).

**Table 4:** Correlations between the plasma levels of APRIL, BAFF, cytokines/chemokines, and parasitaemia in malaria patients in the acute (D0) and convalescent (D15) phases of infection

	Control (n = 12)	*P. vivax* (*n* = 33)		*P. falciparum* (*n* = 19)	
		D0	D15	D0	D15
Variables	*r*	*r*	*r*	*r*	*R*
APRIL versus Parasitaemia	na	−0.144	na	−0.500*	na
BAFF versus Parasitaemia	na	0.104	na	−0.169	na
APRIL versus IFN‐γ	0.214	0.274	0.184	0.174	0.198
APRIL versus TNF‐α	−0.085	0.217	0.158	−0.135	0.105
APRIL versus IL‐1	0.238	0.409*	0.190	0.012	0.023
APRIL versus IL‐2	0.167	0.384*	0.161	−0.105	0.190
APRIL versus IL‐4	0.276	0.397*	0.215	−0.374	0.106
APRIL versus IL‐6	0.126	0.367*	0.123	0.135	−0.139
APRIL versus IL‐8	−0.085	0.189	0.194	0.239	0.225
APRIL versus IL‐10	0.268	0.178	−0.133	−0.202	−0.221
APRIL versus IL‐12	0.167	0.162	0.178	−0.083	0.198
APRIL versus IL‐13	0.262	0.359*	0.105	−0.197	0.177
APRIL versus IL‐17	0.282	0.272	0.191	−0.116	0.159
APRIL versus G‐CSF	0.162	0.140	0.036	−0.003	0.377
APRIL versus MCP1	0.257	0.293	0.149	−0.180	−0.055
APRIL versus MIP1β	−0.200	0.326	0.019	0.106	−0.053
BAFF versus IFN‐γ	−0.123	−0.130	−0.042	0.164	0.321
BAFF versus TNF‐α	0.163	−0.135	0.236	0.125	0.085
BAFF versus IL‐1	−0.285	0.036	−0.139	−0.085	0.085
BAFF versus IL‐2	−0.236	−0.242	−0.033	0.204	0.380
BAFF versus IL‐4	−0.134	−0.112	−0.041	−0.030	0.267
BAFF versus IL‐6	−0.231	0.053	−0.285	0.121	0.121
BAFF versus IL‐8	0.245	−0.052	0.011	0.175	0.082
BAFF versus IL‐10	−0.167	0.198	−0.151	−0.285	0.249
BAFF versus IL‐12	−0.218	−0.040	−0.060	0.133	0.344
BAFF versus IL‐13	−0.294	0.120	0.080	−0.167	0.383
BAFF versus IL‐17	−0.154	−0.281	−0.242	0.367	0.179
BAFF versus G‐CSF	−0.213	−0.020	−0.114	0.257	0.314
BAFF versus MCP1	0.156	−0.012	0.176	−0.070	0.364
BAFF versus MIP1β	0.262	−0.145	−0.339	0.298	−0.309

*r*, Spearman's rank correlation; na, non‐applicable; bold type and *P < 0.05.

### Increased expression of TACI on T cells during the acute phase of *P. vivax* malaria

**Figure 3:**
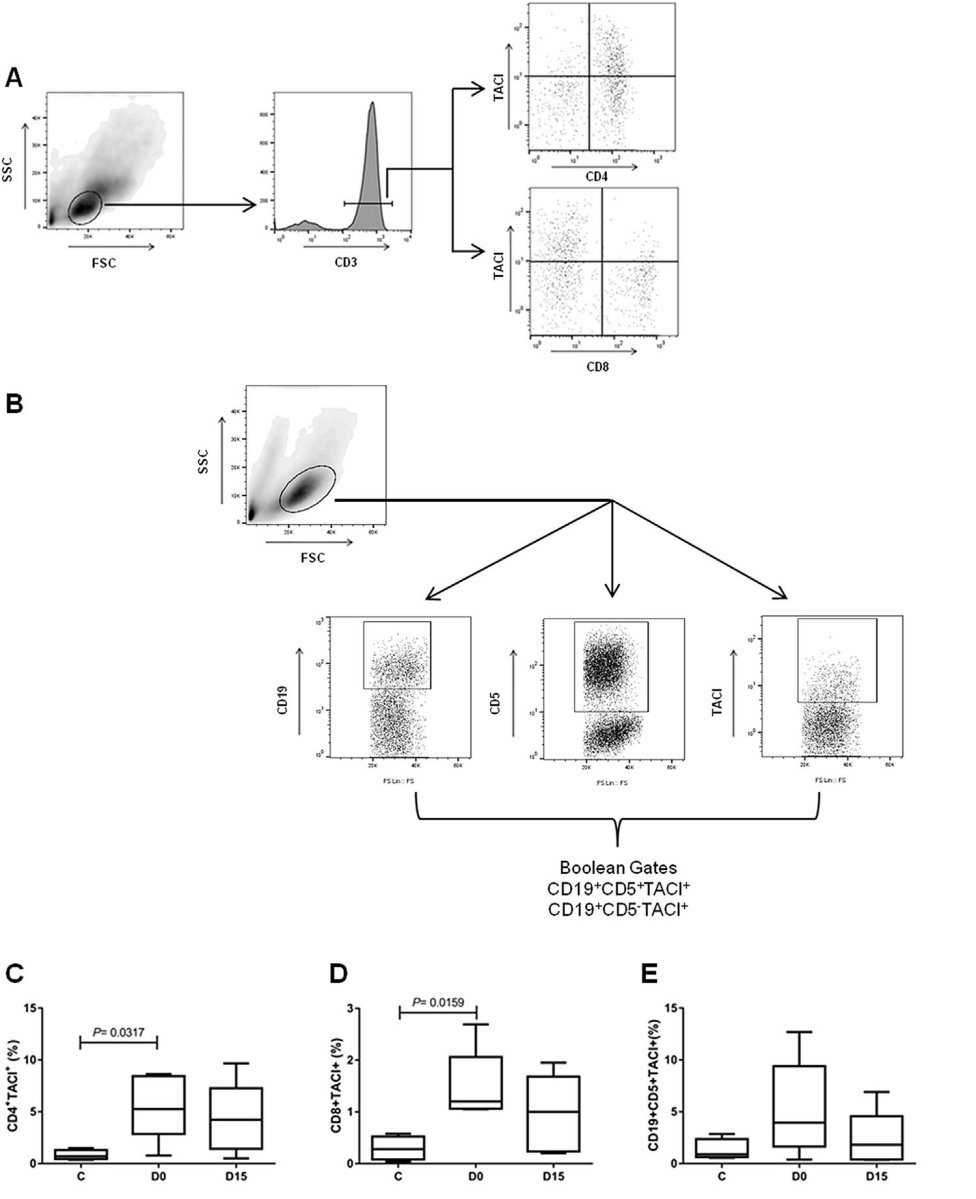
Expression of TACI receptor on T and B cells in malaria patients during acute (D0) and convalescent (D15) phases and in healthy individuals. **A:** Dot‐plot representing gating strategy of CD4+ and CD8+ T cells. A minimum of 30,000 events per sample were acquired inside the lymphocytes gate, based on size and granularity properties (FSC × SSC); followed by selection for CD3+ T cells inside the lymphocytes gate. CD4+ and CD8+ T cells were identified by CD3 and CD4 expression or CD3 and CD8 expression. **B:** Representative dot‐plot of CD19 + CD5 + TACI+ cell. A minimum of 30,000 events per sample were acquired inside the lymphocytes gate, based on size and granularity properties (FSC × SSC); followed by selection of cells expressing CD19, CD5, and TACI inside the lymphocytes gate. We used Boolean Combination Gates toll available in FlowJo software, to perform combinations of the three total surface markers (CD19+, CD5+, and TACI +) to uniquely discriminate CD19 + CD5 + TACI+ cells. **C:** Percentage of CD3 + CD4 + TACI+ cells, D‐CD3 + CD8 + TACI+ cells and E‐CD19 + CD5 + TACI+ cells, observed in the peripheral blood of malarial patients and healthy controls. Wilcoxon matched‐paired test was used to calculate significance levels between the patients (*n* = 5, D0 and D15) and Mann–Whitney unpaired *T*‐test was used to calculate significance levels between patients and control group (*n* = 4).

Transmembrane Activator and CAML Interactor (TACI) is a receptor of the APRIL/BAFF system and is expressed on B cells and activated T cells. To determine whether malaria induces increased expression of TACI, phenotypic analysis of PBMC of five *P. vivax* malaria patients during the acute (D0) and convalescent (D15) phases and four healthy controls was performed (Figs. 3A and 3B). Flow cytometry analysis revealed that the CD4 + TACI+ and CD8 + TACI+ T cells were significantly enhanced in the PBMC of *P. vivax* malaria patients only during the acute phase (D0) compared to the controls (*P* = 0.031 and *P* = 0.015, respectively) (Figs. 3C and 3D). The percentage of the CD19 + CD5 + TACI+ B cell appeared to be enhanced in malaria patients. However, in relation to healthy controls, the differences were not significant in any of the malaria phases (*P* *>* 0.05) (Fig. 3E). No significant differences were observed in the percentage of CD19 + CD5 + TACI+ B cells, CD4 + TACI+ and CD8 + TACI+ T cells in the same samples during acute *versus* convalescent phases of malaria (*P* *>* 0.05, for all).

## Discussion

Malaria is characterized by many pathophysiological changes including alterations in the immune system involving myeloid‐cells, T‐cells and B‐cells. Regarding B‐cells, a number of observations clearly demonstrate that B‐cell homeostasis is affected by *Plasmodium* infection 17, 18, 44, 45. This can be attributed to the complex biology of the parasites 46, 47, their antigenic diversity 46, 47, 48, and their immune‐modulatory molecules 49, 50. The BAFF/APRIL system plays a variety of roles in immunomodulation, mainly by affecting B cell activation, proliferation, and survival. BAFF and APRIL are produced by a range of mononuclear cells such as monocytes, macrophages, neutrophils, dendritic cells, and T‐cells stimulated by cytokines/chemokines often produced during inflammation or infections. Therefore, it is possible that marked changes in cytokine/chemokine levels during malaria 51, 52, 53, as well as *Plasmodium* immune‐modulatory molecules 38 drive BAFF/APRIL production by these cells. In the recent years, many research articles have reported a role for BAFF/APRIL system in the pathogenesis of autoimmune and infectious diseases 23, 26, 31, 32, 33, 34, 35, including malaria 35, 36, 54. However, while there is some evidence that BAFF plays a role in determining the outcome of *P. falciparum* malaria, nothing is known about the role of the follows: 1) APRIL in any malaria or 2) BAFF/APRIL system in *P. vivax* malaria. In this regard, the main objective of this study was to investigate whether BAFF and APRIL plasma concentrations could be part of inflammatory response associated with uncomplicated *P. falciparum* and *P. vivax* malaria in some adults from the Brazilian Amazon that were followed up during the acute and convalescent phases of infection.

First, we observed that the APRIL plasma levels were increased during acute *P. vivax* and *P. falciparum* infections whereas BAFF levels were only increased during acute *P. falciparum* malaria. These findings raise a possibility that the regulation of BAFF secretion may differ between the two strain infections. Although a range of myeloid cells produces BAFF and APRIL, the mechanisms regulating production and expression are poorly understood and likely differ from cell type to cell type. *Plasmodium* spp. infections elicit significant changes including immunological alterations involving myeloid cells and cytokine levels. In line with previous studies 51, 52, 53 we observed marked changes in the cytokine levels during *P. vivax* and *P. falciparum* malaria (Table 3). In both infections, we observed an increase in IFN‐γ, TNF‐α, IL‐6, IL‐8, IL‐10, IL‐13, MIP1β, and G‐CSF levels. However, although *P. vivax* and *P. falciparum* malaria patients have similar cytokine profiles during infection, *P. falciparum* patients presented higher levels of IFN‐γ, TNF‐α, and IL‐2 in acute phase than *P. vivax*. It is known that cytokines such as IFN‐γ, TNF, and IL‐10 as well as G‐CSF, CD40‐L, lipopolysaccharides and peptidoglycans can upregulate BAFF and APRIL expression in different cells types 25, 26, 29, 55, 56, 57. Then, one could speculate that the highest levels of IFN‐γ, TNF‐α, and IL‐2 observed in *P. falciparum*‐infected patients might result directly or indirectly in the upregulation of BAFF production. The increased BAFF plasma levels that we observed during acute *P. falciparum* infection is in accordance with previous findings that showed the elevation of BAFF in plasma from acutely malaria‐infected children and during controlled human malaria challenge (CHMI) in malaria‐naive adults 36, 54 as well as in placental tissue from malaria‐infected pregnant women [37) and on various antigen‐presenting cell subsets 54. Moreover, in vitro, both hemozoin and soluble *P. falciparum* antigens (sPfAg) are capable of inducing BAFF release from monocytes and B‐cell co‐cultures 38. Within this context, it must be evoked that *P. falciparum* and *P. vivax* are genetically distant malaria parasites 58. Probably the heterogeneity between the two species, such as in its chemical composition of soluble antigens 58, 59 and hemozoin 60, 61 could differently modulate BAFF and APRIL production as well as cytokines. Indeed, our findings showed that, in *P. vivax* acute phase malaria, APRIL but not BAFF levels correlated positively with IL‐1, IL‐2, IL‐4, IL‐6, and IL‐13 levels. In addition, we did not find any relationship between *P. vivax* parasitaemia and APRIL levels, while an inverse correlation was found between *P. falciparum* parasitaemia and APRIL plasma levels. Although APRIL and BAFF share several biological characteristics and receptor‐specificity, they have distinct functions. While BAFF is fundamental for B‐cell maturation and survival, APRIL is essential for antibody class‐switching and plasma‐cell survival and is involved in the late stage of B‐cell differentiation 26, 62. Thus, the discrepancies between APRIL and BAFF levels and/or correlations found in this study could also be associated with the different biological functions of APRIL and BAFF during malaria. In addition, we cannot rule out the possibility that genetic polymorphism in the human genes encoding APRIL and BAFF molecules and/or receptors 63, 64, 65, can also play a role in the aforementioned discrepancies.

Consistent with our previous findings 51, the levels of all cytokines increased in the acute phase of both malaria infections and continued to increase after treatment, except for IL‐10. The increased levels of IL‐10 were transient and markedly declined after treatment when parasites were no longer detected. A similar profile was observed for APRIL and/or BAFF levels; the last one is in line with previous findings 36, 54. Thus, plasma APRIL and/or BAFF as IL‐10 levels might reflect the acute phase of malaria. Since IL‐10 upregulates APRIL and BAFF production and secretion 28, 66, 67 and IL‐10 levels correlated positively with parasitaemia, one could speculate that malaria parasites induce an increase of IL‐10 production, which might contribute to the upregulation of APRIL and/or BAFF productions in both infections.

The APRIL and/or BAFF plasma levels are upregulated in many noninfectious 26, 68, 69, 70, 71, 72, 73, 74, 75, 76 and infectious diseases 32, 73, 77, 78, including malaria 36, 54 that are often accompanied by polyclonal B‐cell activation, hypergammaglobulinemia, autoimmune disorders and lymphopenia. In malaria, polyclonal B‐cell activation has been shown in the acute phase of *P. vivax* and *P. falciparum* malaria 15, which decreases 5–15 days after the beginning of treatment 15, with similar transient kinetics of IL‐10, APRIL and/or BAFF levels, observed in our study. Thus, it seems possible that increased IL‐10 levels may contribute to the polyclonal B‐cell activation, via APRIL and/or BAFF upregulation during the acute phase of *P. vivax* and *P. falciparum* malaria.

Consistent with previous findings 51, 79, 80, the lymphocyte counts were significantly decreased during *P. vivax* and *P. falciparum* acute phase malaria. However, lymphopenia was a transient finding and 15 days after the beginning of treatment, the lymphocyte counts were similar to those of control subjects, indicating that this period was sufficient for the patients to achieve lymphocyte homeostasis, according to our previous findings 15. Thus, we believe that APRIL, BAFF, and IL‐10 production might be upregulated as a homeostatic response to the lymphopenia observed in the acute phase of malaria.

TACI is a receptor of the APRIL/BAFF system and is predominantly expressed on B cells and in some subsets of activated T cells 27, 66, 81. Although the spectrum of biological functions of TACI is not fully known, it appears that it plays a crucial role in the antibody response to T cell‐independent and T cell‐ dependent antigens 82, in the induction of plasma cells differentiation and survival 82, in determining macrophage phenotype 83 as well as in promoting division, survival, and activation in T cells 66, 81. To our knowledge, nothing has been published about TACI expression on T cells during human infectious diseases. Regarding B cells, a recent study reported 36 a significantly increased of TACI‐positive B cells during acute and convalescence phases of *P. falciparum* malaria in Kenyan children. We have access to PBMC of five *P. vivax* malaria patients in acute and convalescence phases. Our results showed a transient and significant increase in the percentage of TACI‐positive CD4+ and CD8+ T cells during the acute phase, as well as a transient but not significant increase in the percentage of TACI‐positive CD19+ B cells in *P. vivax* infected donors. Aware that our sample size is too small to make inferences, we also can not rule out the possibility that the discrepancies observed between this study and ours with respect to TACI‐positive B cells, could be associated with the fact that patients involved in our study were adults with uncomplicated *P. vivax* malaria living in different epidemiological scenarios. In relation to T cells, TACI expression presented a transient kinetics similar to IL‐10 and APRIL levels. Thus, the APRIL and IL‐10 plasma levels as well as the proportion of CD4+ and CD8+ T cells expressing TACI might reflect the acute phase of *P. vivax* malaria, and may contribute to calcium‐dependent constitutive activation of nuclear factor of activated T cells (NF‐AT) 84 as observed in the early stage of many infectious diseases 85, 86.

Taken together, we demonstrate that APRIL plasma levels as well as the percentage of TACI positive CD4+ and CD8+ T cells were increased in the acute phase of uncomplicated *P. vivax* infection. Moreover, it seems that the APRIL and BAFF inductions reflect different host strategies for controlling infection with different malaria species, *P. vivax* and *P. falciparum*. Further larger cohort study is ongoing in the Brazilian Amazon to provide better knowledge of the APRIL/BAFF system as well as the TACI B‐ and T‐cell expression in the immunomodulation of malaria.

## Authors' Contributions

RAP performed experiments, statistical analysis and wrote the paper. ACS performed experiments and manuscript review. DSPS performed statistical analysis and manuscript review. RNRS performed experiments. LAS performed experiments. MRA performed statistical analysis. FS did fieldwork support. JCLJ performed collection of blood and epidemiological data and manuscript review. JOF performed collection of blood and epidemiological data. DMVV designed the study and performed manuscript review. PMDL performed the experiments, statistical analysis and manuscript review. CECP designed the study and performed manuscript review. DMB conceived, designed and coordinated the study, performed collection of blood and epidemiological data and wrote the paper. All the authors read and approved the manuscript for publication.

## Conflict of Interest

None.
